# The Impact of Caller Gender on Telephone Crisis-Helpline Workers’ Interpretation of Suicidality in Caller Vignettes

**DOI:** 10.3390/ijerph15040831

**Published:** 2018-04-23

**Authors:** Tara Hunt, Coralie J. Wilson, Peter Caputi, Ian Wilson, Alan Woodward

**Affiliations:** 1 School of Medicine, University of Wollongong, Wollongong, NSW 2522, Australia; cwilson@uow.edu.au (C.J.W); ianwil@uow.edu.au (I.W.); 2 llawarra Health and Medical Research Institute, Wollongong, NSW 2522, Australia; 3 Centre for Mental Illness in Nowra District: Goals and Prevention (MINDtheGaP), Nowra, NSW 2541, Australia; alan.woodward@lifeline.org.au; 4 Centre for Mental Health, University of Melbourne, Melbourne, VIC 3010, Australia; 5 School of Psychology, University of Wollongong, Wollongong, NSW 2522, Australia; pcaputi@uow.edu.au; 6 Lifeline Research Foundation, Lifeline Australia, Canberra, ACT 2601, Australia; 7 Suicide Prevention Australia, Sydney, NSW 2000, Australia

**Keywords:** suicide, suicide intervention, telephone crisis-helpline, telephone crisis support, men, women, communication, gender differences

## Abstract

Telephone crisis-line workers (TCWs) are trained in a variety of techniques and skills to facilitate the identification of suicidal callers. One factor that may influence the implementation of these skills is gender. This study used an experimental design to explore whether helpline callers being identified as male or female is associated with TCWs’ ratings of callers’ potential for suicide risk and TCWs’ intention to use support- or intervention-oriented skills with callers. Data were collected using an online self-report survey in an Australian sample of 133 TCWs. The results suggest that under some circumstances the callers’ gender might influence TCWs’ intention to use intervention-oriented skills with the caller. Implications for the training of telephone crisis workers, and those trained in suicide prevention more broadly are discussed.

## 1. Introduction

Telephone crisis helplines play a pivotal role in comprehensive suicide prevention systems [1]. Each call to crisis helplines provides an opportunity to prevent suicide by facilitating the identification of, and response to, people experiencing imminent suicidal crisis [1,2,3]. Across the USA, UK, Australia and New Zealand, crisis helplines are staffed by paid and unpaid volunteers and para-professionals who are trained in crisis and suicide intervention [4,5,6,7].

**Figure 1:**
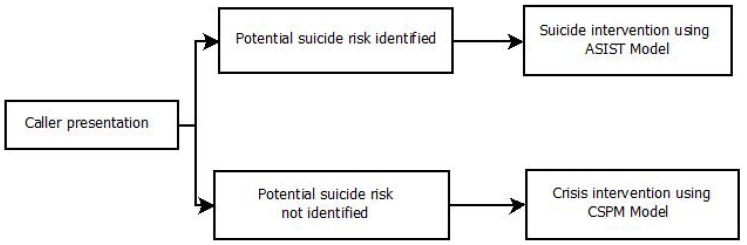
TCW training in decision making.

Training procedures between crisis helplines vary, but across telephone crisis-line services, standardised training protocols tend to apply general guidelines for the identification of callers in a suicidal state and strategies to reduce callers’ current experience of crisis and/or suicidal states. Most also aim to ensure the safety of callers by emphasizing the need to “set aside assumptions about who may be at risk of suicide (usually based on membership in a “higher-risk” group)” [8] and to treat callers as individuals by responding to their unique presentation to identify potential suicidality and direct subsequent support decisions. For example, crisis-line staff (referred to in this article as telephone crisis-line workers: TCWs) at a national telephone crisis helpline in Australia are trained using two intervention models to facilitate efficient and appropriate responses to people in crisis: the Crisis Support Practice Model (CSPM) [9], and the Applied Suicide Intervention Skills Training (ASIST) model [10]. The CSPM guides TCWs’ use of skills involved in connecting with the caller, focusing the call, relieving distress, enabling coping and deciding on next steps. TCWs are trained to monitor every caller’s potential suicidality, regardless of group membership, while progressing through the CPSM by asking about suicidal thoughts in every call. Once the possibility of suicide risk is described by the caller through direct or indirect suicide signs (e.g., callers directly discussing thoughts of suicide, indirect talk of “leaving it all behind”, etc.), TCWs are directed to apply skills from the ASIST model, which are focused on ensuring caller safety [9,11]. In this way TCWs’ training resembles an ‘if–then’ decision model; *if* the possibility of suicide risk is identified as a presenting issue *then* the ASIST model is to be used to specifically check for suicide risk, *if* the possibility of suicide risk is *not* identified, *then* the CSPM is the only model that TCWs implement (see Figure 1).

Current research in clinical decision-making suggests that there is more to decision-making than a straightforward relationship between problem identification and response [12]. Decision-making in healthcare contexts (also known as clinical reasoning or clinical judgement) refers to “the cognitive processes that [are] necessary to evaluate and manage a patient’s…problem” [13]. Sophisticated models of clinical decision-making have been developed for doctors [12], and other health professionals [14,15,16] to specify the mechanisms underlying decision-making and understand how errors occur. Such models propose that decision-making can be influenced by a variety of contextual factors, state factors, and biases. For example, mental workload and fatigue can impair information processing accuracy and efficiency, and consequently contribute to poor performance on decision-making tasks [17,18]. Additionally, negative psychological states can be associated with deficits in the ability to infer others’ mental states and predict their behavior [19], and may impair decision-making and help-provision in occupational contexts [20]. In the telephone crisis support context, these factors may influence TCWs’ identification of potential suicide risk. At the national telephone crisis helpline in Australia, TCWs are trained to listen for whether a caller might be suicidal but *not* to clinically assess for the presence of suicide risk. If callers do not describe suicidal thoughts in a direct way, which may be up to two-thirds of people with thoughts of suicide [21], it is up to the TCW to infer whether suicidal risk *could* be present and the potential degree of severity, based on the TCW’s recognition of signs of suicide that may be described by the caller. This decision-making process involves pattern recognition that is defined as the “non-conscious recognition of problem-states based on patterns of features that prime appropriate scripts in memory” [22].

There is substantial evidence to suggest that when TCWs are required to infer whether an ambiguous caller could be suicidal, TCWs will be influenced more by heuristics and biases than other factors that underpin decision-making [12,23,24]. A prominent factor that may bias the inference of suicidal presentation is the caller’s gender [25]. Gender refers to a system of social relations and practices that assign people to one of two categories (male or female) [26,27] that define the “differing characteristics of men and women and how [each group is] *expected* to behave” [27] (p. 512, emphasis added). Across Western countries, statistical data indicates that men are over-represented in suicidal fatalities, and women are over-represented in suicide attempts [28,29,30]. Some suicide intervention training programs such as ASIST caution helpers to not apply risk-group categorization to individual cases as it only reflects population differences in suicidal vulnerability, and may result in suicidal individuals being missed [8]. Yet, it is possible that in the absence of information that clearly describes a caller’s suicidal state, TCWs may be influenced by their perception of the callers’ gender when making sense of the information they are hearing. For example, there is emerging evidence that when TCWs are deciding whether a caller might be suicidal, they listen for patterns of information that are subtly different in male and female callers [25]. If TCWs listen for different patterns of suicide signs for male and female callers, it is also possible that a caller’s gender will influence TCWs’ decision to prioritise their use of skills associated with the CSPM model (support-oriented skills) or the ASIST model (intervention-oriented skills). Whether there is such a relationship between callers’ gender and TCWs’ decision to use one type of skills over the other is currently unknown. Given the lethal consequences that may be associated with inaccurate decision-making with suicidal callers, a better understanding of whether a gender bias might influence TCWs’ decision-making may help save a life.

The current study used a repeated-measures experimental design to explore whether there is an association between TCWs’ ratings of callers’ potential for suicide risk and their intention (i.e., the action component of the decision to act; [31]) to use intervention-oriented or support-oriented skills with male and female callers, with contextual factors (e.g., TCW’s recent shift workload; TCW’s own gender) and state factors (e.g., TCW’s current psychological state) controlled for.

## 2. Methods

### 2.1. Participants

One hundred and forty-eight participants were recruited from an Australian national crisis helpline and were all trained in the CSPM and ASIST intervention models. ASIST is a 14-h internationally recognized gatekeeper training program [10] that is delivered alongside the CSPM model [9] that was developed by the helpline as a part of its standardized training procedures. Phase 1 of TCW training involves a minimum of 67 contact hours over 3 months, which covers common caller presenting issues (e.g., grief and loss, relationships, drug and alcohol issues, suicide, etc.). The sessions are facilitated by two trainers, and usually involve lectures followed by role-plays to practice applying the service delivery models to a variety of caller situations. Completion of Phase 1 of standardized training prior to answering calls on the crisis line is mandatory for all TCWs. After completing Phase 1, TCWs proceed to Phase 2 and 3 which involves supervised call shifts and ongoing call assessment to ensure competency and consistency in delivering the service provision models.

Fifteen TCWs completed the demographic section and exited the survey prior to completing the study measures, and their demographic data were excluded from analysis. Sample age was representative of the National TCW population based on figures provided by the crisis helpline, and ranged from younger than 25 years to older than 66 years; 16.5% of the sample (*n* = 22) was younger than 35 years, 66% (*n* = 91) was aged 36 to 65 years, and 12.8% (*n* = 20) was aged 66 years or older. The largest proportion of the sample, 48.1% (*n* = 64), had been a TCW for 0 to 2 years; 36.1% (*n* = 48) had been a TCW for 3 to 8 years and 15.8% (*n* = 21) had been a TCW for 9 years or more. Most in the sample, 97.1% (*n* = 129), reported that they completed shifts at least once per fortnight, and 3.0% (*n* = 4) completed shifts once per month. Most in the sample, 85% (*n* = 113), also reported that they had completed their last shift in the past fortnight, whereas 9.8% (*n* = 13) completed their last shift 3 to 4 weeks ago and 5.4% (*n* = 7) completed their last shift more than 4 weeks ago. Additional sample context information is presented in Table 1.

### 2.2. Design

**Figure 2:**
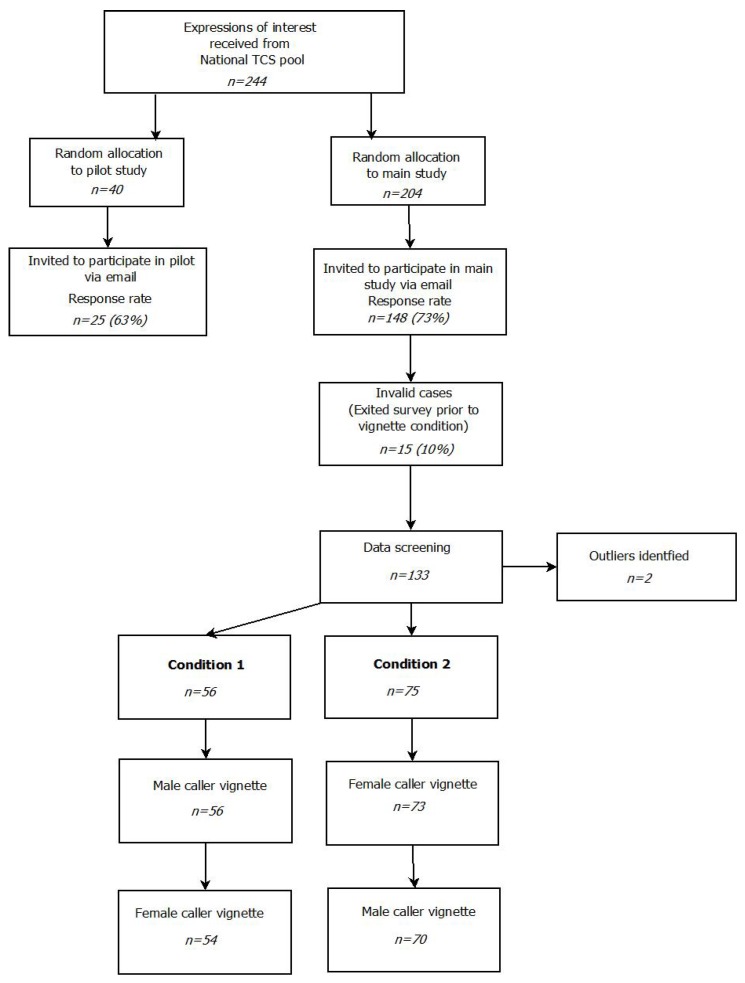
Recruitment flow diagram for pilot and main study.

**Figure 3:**
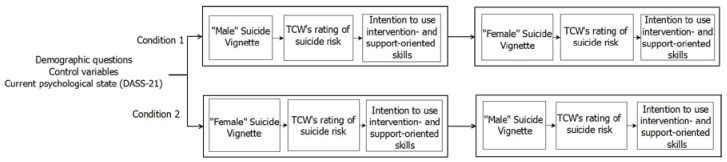
Study design exploring the association between Telephone Crisis Workers’ (TCWs’)TCWs’ rating of callers’ potential for suicide risk and their intention to use intervention- and support-oriented skills with male and female callers.

The study design and procedure was developed within the context of a community-academic partnership (CAP) using a co-design process [32] (a detailed description of the CAP and project co-design can be found elsewhere [33]). The co-design process utilized a structured approach to seeking feedback from different levels of the crisis helpline to develop a design approach that was suitable for the context. Since the impact of gender on TCWs’ support of suicidal callers has yet to be examined, an experimental design wherein gender was manipulated was nominated as the most prudent approach. Such a design would allow the initial exploration of the impact gender may have on the process of help-provision compared to the baseline provided by TCWs’ standardized training. The feedback process identified that it was a priority to develop a design that was as succinct as possible for participants who were predominantly volunteer TCWs with high workloads. As a result, this study used an online self-report survey in a repeated-measures vignette design to systematically examine whether caller gender might influence the information that TCWs identify as important when deciding whether a caller might be suicidal and whether to prioritise the use of support-oriented or intervention-orientation skills with the caller. To implement the design, participants were randomly assigned to one of two groups and exposed to both study conditions which used the same caller vignette with different names and gender pronouns (see Figure 2; more detail below). The order of vignette presentation was counter-balanced to examine whether the gender of the caller initially presented might influence the information TCWs attended to throughout the study survey Figure 3). Repeated-measures designs with the use of vignettes have been used successfully, with minimal evidence of evaluation effects that impair interpretation of study results [34].

#### Vignette Justification and Development

Collecting data by listening to actual crisis-line calls raises ethical issues related to caller privacy, confidentiality, and ability to provide valid and informed consent due to the vulnerability of callers that must be considered against study merit when conducting in vivo studies [35]. Since there are no existing studies that have explored whether callers’ gender biases TCWs’ decision-making and intention to use support- or intervention-oriented skills with suicidal callers, this study used a standardised vignette, rather than an in vivo setting, to explore whether such a bias might exist.

An ambiguous caller vignette was drafted by the research team using commonly recognised signs of suicide [36], and was distributed among the clinical training staff at the telephone helpline for feedback about face- and content-validity. Each suicide sign identified by Rudd et al. [36] was described in the vignette but there were no direct statements of suicidal intent. The gender of the caller that is described in the vignette was manipulated by changing the name and gender pronouns to either Jack and he/his, or Jill and she/her. Changing gender pronouns that are used in vignettes has been found to trigger responses that are informed by gender [37,38,39]. The vignette read: (Jill/Jack) has called a helpline. (S/he) is feeling trapped. In the past few months (Jill/Jack) has experienced a relationship breakdown, which (s/he) continues to feel angry about. Currently (Jill/Jack) seems to be agitated and restless. (S/he) says (s/he) wants to get in (his/her) car, drive away and leave everything behind. Most nights (Jill/Jack) has trouble sleeping. Recently (s/he) has been having a few drinks before bed as a way to relax and fall asleep. (Jill/Jack) doesn’t feel like leaving the house, and often avoids social contact. (Jill/Jack’s) family and friends have noticed this change, and are becoming concerned about (his/her) changes in mood. Jill/Jack says called the helpline because (he/she) doesn’t see things ever improving. Based on the suicide risk assessment guidelines that this sample of TCWs were trained to follow, the appropriate response to a caller who might be suicidal, but who has not made a direct expression of suicidal intent, is to follow the general CSPM model which advises the use of support-oriented skills until the TCW conducts a safety check and the caller’s suicidal intent is confirmed or disproved.

### 2.3. Recruitment

The study protocol was approved by the University of Wollongong Human Research Ethics Committee (16/135) and UnitingCare Queensland Human Research Ethics Committee (Wilson C.19016). After approval from relevant ethics review boards, Telephone Crisis Support Centre Managers who consented to allow TCWs from their centres to participate in the study were asked to distribute an online expression of interest form among active TCWs. Participants were told that the research required completing an online survey that included questions about TCWs’ decision-making with callers. The study focus on suicidal callers was not explicitly mentioned. The TCWs who expressed interest in participating in the research project were approached only once by the research team and those who provided consent to participate in the study were assigned to either the pilot or main study (see Figure 3).

### 2.4. Procedure

Participants who were assigned to the pilot study and provided consent completed the study survey before those who were assigned to the main study. The purpose of the pilot study was to determine whether the design and survey were feasible and appropriate within a TCW sample. Feedback was sought from participating TCWs by an open-response comment and feedback section at the conclusion of the pilot survey. Additionally, managers of the participating helpline centers were called prior to and during survey distribution to collect informal feedback. The response rate for the pilot study was 62.5% (*n* = 25). The findings from the pilot study led to small adjustments to the phrasing of two survey questions and clarification of the purpose of the survey in the Participant Information Sheet that introduced and described the study to participants.

This article reports results from the main study. The response rate for the main study survey was 73% (*n* = 148). An independent survey administrator who was not a part of the research team communicated directly with participating TCWs via email. This strategy assured the confidentiality and anonymity of research participants. Participants who were allocated to the main study were sent an electronic link to the Participant Information Sheet, which included an electronic button to provide consent. TCWs who provided consent were redirected to the study survey hosted by SurveyMonkey©. The first section of the survey included demographic questions and the study control variables (psychological state, mental workload, and gender identity), and was completed by all participants prior to being assigned to a Condition. Participants were then randomly allocated to Condition 1 or Condition 2 using the randomization function in SurveyMonkey©, which resulted in sample sizes of 56 and 75, respectively. Participants in Condition 1 were asked to read a vignette describing a suicidal male caller, rate the possible suicide risk of the caller, and report intention to use intervention- and support-oriented skills with the caller. Once participants in Condition 1 had completed this section, they were presented with the vignette describing a suicidal female caller, and responded to the same questions. Participants in Condition 2 were asked to read a vignette describing a female caller, rate the possible suicide risk and report intention to use intervention- and support-oriented skills with the caller. Once participants in Condition 2 had completed this section, they were presented with the vignette describing the suicidal male caller and asked to respond to the same questions. Participants were thanked for their participation and then exited the survey.

### 2.5. Measures

*Demographics*. All participants completed questions that asked for gender, age, home location, country of birth, and years of experience as a TCW. TCWs also reported whether they had lived experience of suicide, defined as “having experienced suicidal thoughts, survived a suicide attempt, cared for someone who has attempted suicide, been bereaved by suicide, or been touched by suicide in another way” [40].

*Workload*. Participants reported how often they complete shifts at the telephone helpline (shift frequency) and when they completed their most recent shift at the helpline (shift recency) on a scale from 1 Less than one week ago to 6 More than 4 weeks ago. Shift frequency and shift recency were correlated (*r* = 0.44, *p* < 0.001), and were used to create a composite workload measure by combining and averaging z-scores.

*Current psychological state.* The 21-item Depression, Anxiety, Stress Scales (Lovibond & Lovibond, 1995: DASS-21) is the short-form of the 42-item DASS and contains three scales of 7 items that measure the negative emotional states of depression, anxiety and stress. Sample items include “I felt that I had nothing to look forward to”, “I felt I had a lot of nervous energy”, and “I tended to over-react to situations”, and are rated from 0 (Did not apply to me at all) to 3 (Applied to me very much, or most of the time). The DASS-21 has high levels of internal reliability for each scale: depression (α = 0.90), anxiety (α = 0.79), and stress (α = 0.89) [41]. For analysis, a composite measure was calculated by summing the raw scores from each scale to generate a measure of general psychological distress [41].

*Rating of callers’ potential suicide risk.* TCWs were presented with a vignette of a male or female caller presenting signs of suicide. After reading the vignette, TCWs were asked to rate the likelihood that the caller in the vignette was suicidal. TCWs responded to a single item, “In your opinion, what is the suicide risk of this caller” by selecting “low” (1), “medium” (2) or “high” (3). Few TCWs rated the caller as “low” risk (1% to 2% of rankings across vignette presentations), thus, “low” and “medium” rankings were summed to create a single “low–medium” category score for analysis (see Table 2).

*Intention to use TCW skills.* The Telephone Crisis Support Skills Scale (TCSSS) [42] was used to measure TCWs’ intentions to use the recommended TCW service skills in response to the vignettes of suicidal crisis-line callers. The 23 items in the TCSSS were taken directly from the standardized training protocol that all TCWs in this sample received before taking crisis calls. Items assess intention to use either the support-oriented or intervention-oriented skills that are itemised in the CPSM and ASIST intervention models. Participating TCWs were asked to rate their intention to use each skill with the caller described in the vignette on a scale from 1 (extremely unlikely to use) to 4 (extremely likely to use). For analysis, responses to skills were grouped as support-oriented or intervention-oriented, creating two subscales corresponding to the specific content of the CSPM and ASIST models. Sample support-oriented items include “Identify what prompted the call/main issue”, and “Engage the caller’s trust”. Sample intervention-oriented items include “Increase safety” and “Manage the immediate situation”. The subscales contained 13 support-oriented skills (α = 0.93) and 10 intervention-oriented skills (α = 0.89) that demonstrated acceptable levels of internal consistency.

### 2.6. Statistical Analysis

A priori power estimates were calculated with G*Power (University of Düsseldorf, Düsseldorf, Germany) [43] to identify the number of participants required to find a small effect size. Making the assumption of a β level of 0.85 and alpha of 0.05, it was found that the study would require a minimum of 50 participants per condition to detect a small effect.

Prior to analysis, scores for the TCSSS, DASS, and rating of possible suicide risk items were examined in SPSS (IBM, Armonk, NY, USA), and univariate and multivariate outliers, and non-normal distributions were also screened [44]. Visual inspection of normal Q–Q plots suggested that the measures were approximately normally distributed and multiple regression was deemed appropriate for use with the data. Outliers across the conditions were examined, and two cases were excluded that had unacceptably high standardised residuals (>3), leverage values across conditions (>0.2) and were influential points (Cook’s Distance value greater than 1), leaving 56 participants in Condition 1 and 75 participants in Condition 2.

Frequencies were calculated of the TCW’s rating of callers’ potential suicide risk along with means and standard deviations for each remaining study variable (age, gender, and years of TCW experience). To explore whether the gender of the caller vignette, or the order of vignette presentation, impacted the frequencies of TCWs’ rating of callers potential suicide risk, a chi-squared test of independence was calculated using an alpha level of 0.05. Bivariate correlations between study measures, within each Condition, were calculated.

Prompted by either the male or female caller vignette, a series of multivariate regression models was used to examine whether TCWs’ intention to use intervention- and support-oriented skills with male or female suicidal callers was associated with rating of possible suicide risk with context (current psychological state, shift load and TCWs’ gender) controlled for. Eight models were run: four for each condition with the first and second models (Condition 1) prompted by the male caller vignette, the third and fourth models (Condition 1) prompted by the female caller vignette, the fifth and sixth models (Condition 2) prompted by the female caller vignette, and the seventh and eighth models (Condition 2) prompted by the male caller vignette. The Bonferroni-adjusted alpha for multiple calculations was 0.006. For the analysis, the alpha value was manually set at 0.01 as the Bonferroni correction has been critiqued for being a conservative adjustment [45,46]. To account for the presence of potential order effects, regression models were run separately on split file data for participants in Condition 1 (*n* = 56) and Condition 2 (*n* = 75). In models one, three, five and seven, intention to use support skills was used as the dependent variable(DV) and in models two, four, six and eight, intention to use intervention skills was used as the DV. In all eight regressions, shift load was entered as the first IV, followed by current psychological state, TCWs’ gender, and TCWs’ rating of callers’ potential suicide risk.

## 3. Results

### 3.1. Descriptives

Frequencies of the TCWs’ ratings of callers’ potential suicide risk are reported in Table 2. Between 41% and 56% of the sample rated the male and female caller vignettes as low-medium risk, whereas 44% to 59% of the sample rated the male and female caller vignettes as high risk (Table 2). Chi-squared analyses were used to identify whether there were differences between TCWs’ rating of male and female callers suicide risk across Condition 1 and Condition 2. The analyses found that the potential suicide risk ratings for male and female callers were not significantly different.

Means and standard deviations for all other measures are reported with reliability estimates in Table 3. TCWs’ reported an average level of general psychological distress that is expected to be lower than 63% of the general Australian population (percentile rank = 37; 95% CI = 33 to 40) [41,47]. TCWs also reported that they were *likely* to use TCW skills with male (*M* = 3.60, *SD* = 0.41) and female callers (*M* = 3.66, *SD* = 0.03). The reliability estimates for all multi-item scales were found to be acceptable (α ˃ 0.70; [48]).

Intention to use support-oriented skills and intention to use intervention-oriented skills were correlated with age, gender, lived experience of suicide and years of TCW experience within each Condition. Correlations that were significant at 0.05 are reported below.

In Condition 1 (male-female vignette presentation), TCWs’ intention to use intervention-oriented skills with the male caller was associated positively with TCWs’ rating of the male callers’ potential suicide risk, *r* = 0.51, *p* < 0.001. Intention to use intervention-oriented skills with the female caller was associated positively with TCWs’ rating of female callers’ potential suicide risk, *r* = 0.37, *p* = 0.006. TCWs’ intention to use support-oriented skills with the male caller was associated positively with TCWs’ rating of the male callers’ potential suicide risk, *r* = 0.35, *p* = 0.008.

In Condition 2 (female-male vignette presentation), TCWs’ intention to use intervention-oriented skills with the male caller was associated positively with TCWs’ rating of the male callers potential suicide risk, *r* = 0.24, *p* = 0.049.

### 3.2. Association between Potential for Suicide Risk and Intention to Use Support or Intervention Skills

#### 3.2.1. Male-Female Vignette Presentation (Condition 1)

In Condition 1, regression models one and three (DV: intention to use support skills) were not significant (model 1: *R*^2^ = 0.15, Adj *R*^2^ = 0.09, *F*(4, 50) = 2.27, *p* = 0.075; model 3: *R*^2^ = 0.11, Adj *R*^2^ = 0.04, *F*(4, 48) = 1.50, *p* = 0.216) and there were no significant associations with intention at α = 0.01 within either model (Table 4). Models two and four (DV: intention to use intervention skills) were both significant (model 2: *R*^2^ = 0.28, Adj *R*^2^ = 0.23, *F*(4, 50) = 4.95, *p* = 0.002, Cohen’s *f*^2^ = 0.38; model 4: *R*^2^ = 0.24, Adj *R*^2^ = 0.18, *F*(4, 48) = 3.85, *p* = 0.009, Cohen’s *f*^2^ = 0.31) and TCWs’ rating of callers’ possible suicide risk was associated significantly with TCWs’ intentions to use intervention skills with the male caller and the female caller (Table 4). By Cohen’s (1988) conventions, an effect of this magnitude for models two and four can be considered “large” and “medium”, respectively [49].

#### 3.2.2. Female-Male Vignette Presentation (Condition 2)

In Condition 2, regression models five and seven (DV: intention to use support skills) were not significant (model 5: *R*^2^ = 0.09, Adj *R*^2^ = 0.04, *F*(4, 66) = 1.67, *p* = 0.166; model 7: *R*^2^ = 0.02, Adj *R*^2^ = −0.04, *F*(4, 65) = 0.32, *p* = 0.866), and models six and eight were not significant (model 6: *R*^2^ = 0.11, Adj *R*^2^ = 0.05, *F*(4, 66) = 1.95, *p* = 0.113; model 8: *R*^2^ = 0.07, Adj *R*^2^ = 0.01, *F*(4, 65) = 1.23, *p* = 0.306). Consistent with models one and three in Condition 1, there were no significant associations with intention found within any model run within Condition 2 (Table 4).

## 4. Discussion

This study explored whether TCWs’ ratings of callers’ potential for suicide risk are associated with their intention to use intervention-oriented or support-oriented skills with male and female callers. It was found that when presented with vignettes of male and female callers describing the same signs of suicide, TCWs rated the male and female caller as experiencing similar potential for suicide risk. The study also found that TCWs’ ratings of callers’ potential for suicide risk were associated significantly with their intention to use intervention skills with male and female callers, but only when TCWs initially responded to the vignette describing a suicidal male caller (Condition 1). When TCWs were presented with the male caller before the female caller (Condition 1), the association between TCWs’ rating of potential suicide risk with their intention to use support-oriented skills with the female caller was not significant. In contrast, in Condition 2, when TCWs were presented with the female caller before the male caller, TCWs’ rating of potential for suicide risk was not associated significantly with either type of intention. These results suggest that consistent with their suicide intervention training, TCWs recognize suicidal potential in both male and female callers expressing signs of suicide. However, the finding that TCWs’ rating of the male callers’ possible suicide risk was associated with intention to use intervention-oriented skills in Condition 1 suggests that TCW decision-making might be influenced by biased by caller gender.

The finding that TCWs rated the male and female caller in each vignette at similar levels of potential suicide risk is consistent with existing studies that have found that similar levels of risk are associated with the same signs of suicide in men and women [50]. Although emerging research suggests that TCWs may recognize potential for suicide risk through different patterns of suicide signs for male and female callers [25], the current study suggests that a caller’s gender may not influence TCWs’ perception of the potential suicide risk posed by the caller. This finding suggests that the suicide intervention training procedures that were used with this sample of TCWs—procedures that emphasise the necessity of acknowledging and responding to all suicide signs expressed by callers—appear to be effective at ensuring optimal responses to suicidal presentation, regardless of a caller’s gender [10].

Although potential suicide risk was identified consistently across male and female callers, in this study, TCWs’ rating of callers’ potential suicide risk was only associated with intention to use intervention-oriented skills when the initial vignette was male, not female. This finding suggests that TCWs may be biased when supporting men who are possibly suicidal, and adopt additional vigilance and caution by associating signs of suicide with the need to defer to intervention-oriented skills. This contrasts with the suicide risk assessment guidelines that the current sample of TCWs were trained to follow (described in the introduction) which suggests that the appropriate response to a caller who expresses signs of suicide, but has not directly indicated they are suicidal, is use of support-oriented skills. It also contrasts with the service standard for TCWs to not modify their response to callers based on perceived risk-group membership. Yet, the TCWs’ response to the suicidal male caller in Condition 1 may be appropriate. Men are overrepresented in suicide mortality statistics across the Western World [30], and in Australia, where the crisis helpline is based, the suicide rate for men is three times higher than that for women [51]. It is possible that TCWs learn to associate male suicidal presentation with the need for intervention skills as a result of the pairing of men and suicidal mortality that occurs in media reporting, print media, television and film depictions, and from informal conversations that occur within the standardized training context with peers and trainers [52,53,54,55].The results of this study suggest that TCWs are flexible in their adherence to the service delivery, and the inferred gender of a caller may trigger slightly different decisions about when to apply intervention-oriented skills once potential suicidality is identified. A caution to this finding is that if TCWs always respond to male callers who are potentially suicidal with intervention-oriented skills they might select skills that are not best suited to the callers’ needs. For example, if TCWs automatically use intervention skills when encountering a male caller whom they perceive to be at high risk, there is a possibility that male callers’ needs for emotional support may be overlooked [56]. Additionally, female callers at risk of suicide may be overlooked. Consequently, TCWs require the process skills and awareness to ensure that decisions to apply one service model over the other are made in a cautious and deliberate way rather than as an automatic reflex.

The TCWs’ largely consistent pattern of responses speaks to the effectiveness of the standardized suicide intervention program the current sample was trained in. However, the finding that there may be conditions under which the TCWs’ responses are impacted by the callers’ gender suggests that current training practices may be enhanced. Firstly, suicide intervention training could be supplemented with additional information explaining how experiences and representations of suicidality can be gendered, and the ways this may impact helpers’ behavior. Secondly, the finding that TCWs may unconsciously adapt their use of the service provision models based on their knowledge of the association between men and suicide suggests that suicide intervention training organizations may need to consider this in designing programs. TCWs may be supported in individualized approaches to suicide prevention with training in decision-making to mitigate potential biases, and assist helpers in using their own knowledge and experience of suicide in a helpful and consistent way. The inclusion of cognitive-debiasing would involve increasing TCWs’ awareness of potential biases that can exist in decision-making and developing the knowledge and strategies needed to overcome these biases under different conditions with callers [57,58,59]. Together the findings from this study highlight the need for ongoing training that strengthens TCWs’ competence in responding to callers’ separate and unique needs, regardless of expectations of suicidal presentation between various groups that are perpetuated through scientific research and the media.

Interpretation of these results should also take into account limitations. The repeated-measures study design that allowed the systematic manipulation of gender in caller vignettes may have introduced order effects into TCWs’ pattern of responses. The study mitigated the impact of this on the results by not directly comparing TCWs’ responses to the male and female vignettes between conditions, but instead was interested in how the order effects differed between conditions. Future research should explore whether the study findings can be replicated in different caller scenarios, and in an in vivo context. While vignettes do not completely recreate the context of a live call, they do provide an opportunity to examine participants’ schematic knowledge of previous calls and TCWs’ response patterns that are stored in their memory [60,61]. Reading caller vignettes triggers TCWs’ autobiographical memories of caller cases that had similar presenting features to the vignette. These memories trigger how the TCWs are likely to respond to a future caller and therefore provide an authentic reflection of real-life decision-making patterns [61,62]. This exploratory study has found that there may be situations in which the callers’ gender impacts TCWs’ decisions to use particular types of skills they have been trained in. However, these results likely reflect a combination of training as well as context and/or state variables, and it is unclear the extent to which each influences the findings. Future research should investigate the ways in which TCWs’ context and state impacts decision-making with callers and the conditions under which gender bias is most likely to impact caller care in a more naturalistic setting.

## 5. Conclusions

The findings provide evidence to suggest that TCWs’ decision-making with suicidal callers may be influenced, to some extent, by caller gender. This is the first time that the influence of gender on the interpretation and response to suicidal presentation has been examined, and has implications for the training of telephone crisis supporters, and those trained in suicide prevention more broadly. The finding that the gender of the initial caller in a series of calls triggered different decisions for using trained skills in response to potential suicidality suggests that gender may impact the process of suicide intervention in ways that have not previously been considered. Subject to further research, the results of this study suggest that suicide intervention training for TCWs and other front-line responders may be enhanced by considering factors that influence the interpretation of and response to people with thoughts of suicide.

## Figures and Tables

**Table 1:** Sample information.

Demographics	Range	*n*	%
Home location	Metropolitan	78	58.6
	Regional	45	33.8
	Rural/Remote	10	7.5
Lived experience of suicide	Yes	95	71.4
	No	37	27.8
	Prefer not to respond	1	0.8
Lifeline position	Volunteer	121	84.2
	Paid	21	15.8

**Table 2:** Frequencies and percentages of TCWs’ rating of callers’ potential for suicide risk by vignette condition.

**Male-Female Vignette Order (Condition 1)**	**Possibility of Suicide Risk**	**Male Vignette Frequency (Percentage of Condition 1 Sample)**	**Female Vignette Frequency (Percentage of Condition 1 Sample)**
	Low–medium	23 (41%)	29 (53%)
	High	33 (59%)	26 (47%)
**Female-Male Vignette Order (Condition 2**)	**Possibility of Suicide Risk**	**Female Vignette Frequency (Percentage of Condition 2 Sample)**	**Male Vignette Frequency (Percentage of Condition 2 Sample)**
	Low–medium	41 (56%)	30 (43%)
	High	32 (44%)	40 (57%)

**Table 3:** Descriptive statistics and reliability estimates of study scales.

Scale	*M*	*SD*	No. of Items	α
Current psychological state (Depression, Anxiety, Stress Scale-21)	6.96	0.53	21	0.91
*Male-female vignette* (*Condition 1*)	7.16	0.94		
*Female-male vignette* (*Condition 2*)	6.61	0.66		
Workload (composite)	0.00	0.14	1	N/A
*Male-female vignette* (*Condition 1*)	0.02	0.22		
*Female-male vignette* (*Condition 2*)	−0.25	0.19		
Intention to use skills with male caller	3.60	0.41	23	0.96
Intention to use support-oriented skills	3.70	0.47	13	0.95
*Male-female vignette* (*Condition 1*)	3.72	0.06		
*Female-male vignette* (*Condition 2*)	3.69	0.06		
Intention to use intervention-oriented skills	3.49	0.53	10	0.91
*Male-female vignette* (*Condition 1*)	3.56	0.07		
*Female-male vignette* (*Condition 2*)	3.42	0.65		
Intention to use skills with female caller	3.66	0.03	23	0.94
Intention to use support-oriented skills	3.76	0.36	13	0.80
*Male-female vignette* (*Condition 1*)	3.72	0.06		
*Female-male vignette* (*Condition 2*)	3.79	0.03		
Intention to use intervention-oriented skills	3.54	0.46	10	0.83
*Male-female vignette* (*Condition 1*)	3.55	0.07		
*Female-male vignette* (*Condition 2*)	3.53	0.05		

**Table 4:** Results of multivariate regression models examining the association between TCWs’ rating of callers’ potential for suicide risk and their intention to use support or intervention skills with male and female callers.

**Independent Variable** ^**a**^	**Condition 1**							
	**Male Vignette** ^**b**^				**Female Vignette** ^**c**^			
	Model 1: Intention to use support skills				Model 3: Intention to use support skills			
	B	β	*sr* ^2^	*p*	B	β	*sr* ^2^	*p*
Possible suicide risk	0.17	0.32	0.33	0.02	0.11	0.24	0.25	0.08
	**Male Vignette** ^**b**^				**Female Vignette** ^**c**^			
	Model 2: Intention to use intervention skills				Model 4: Intention to use intervention skills			
	B	β	*sr* ^2^	*p*	B	β	*sr* ^2^	*p*
**Possible suicide risk**	**0.36**	**0.49**	**0.49**	**0.00**	**0.31**	**0.39**	**0.41**	**0.00**
	Condition 2 ^c^							
	**Female Vignette** ^**d**^				**Male Vignette** ^**e**^			
	Model 5: Intention to use support skills				Model 7: Intention to use support skills			
	B	β	*sr* ^2^	*p*	B	β	*sr* ^2^	*p*
Possible suicide risk	−0.02	−0.04	−0.04	0.77	0.09	0.09	0.09	0.49
	**Female Vignette** ^**d**^				**Male Vignette** ^**e**^			
	Model 6: Intention to use intervention skills				Model 8: Intention to use intervention skills			
	B	β	*sr* ^2^	*p*	B	β	*sr* ^2^	*p*
Possible suicide risk	0.13	0.17	0.17	0.16	0.27	0.24	0.23	0.06

^a^ Workload, current psychological state, and TCS gender controlled for, ^b^ *n* = 56, ^c^ *n* = 54, ^d^ *n* = 73, ^e^ *n* = 70,
